# Invasibility of a North American soil ecosystem to amphibian-killing fungal pathogens

**DOI:** 10.1098/rspb.2023.2658

**Published:** 2024-04-17

**Authors:** Sarah E. McGrath-Blaser, Natalie McGathey, Allison Pardon, Arik M. Hartmann, Ana V. Longo

**Affiliations:** Department of Biology, University of Florida, Gainesville, FL 32611, USA

**Keywords:** *Batrachochytrium dendrobatidis*, *Batrachochytrium salamandrivorans*, invasion, fungi, Smoky Mountains

## Abstract

North American salamanders are threatened by intercontinental spread of chytridiomycosis, a deadly disease caused by the fungal pathogen *Batrachochytrium salamandrivorans* (*Bsal*). To predict potential dispersal of *Bsal* spores to salamander habitats, we evaluated the capacity of soil microbial communities to resist invasion. We determined the degree of habitat invasibility using soils from five locations throughout the Great Smoky Mountains National Park, a region with a high abundance of susceptible hosts. Our experimental design consisted of replicate soil microcosms exposed to different propagule pressures of the non-native pathogen, *Bsal*, and an introduced but endemic pathogen, *B. dendrobatidis* (*Bd*). To compare growth and competitive interactions, we used quantitative PCR, live/dead cell viability assays, and full-length 16S rRNA sequencing. We found that soil microcosms with intact bacterial communities inhibited both *Bsal* and *Bd* growth, but inhibitory capacity diminished with increased propagule pressure. *Bsal* showed greater persistence than *Bd*. Linear discriminant analysis (LDA) identified the family Burkolderiaceae as increasing in relative abundance with the decline of both pathogens. Although our findings provide evidence of environmental filtering in soils, such barriers weakened in response to pathogen type and propagule pressure, showing that habitats vary their invasibility based on properties of their local microbial communities.

## 1. Introduction

Microbial invasions significantly contribute to global diseases of plants, animals and humans, with profound impacts on community and ecosystem health [1–3]. Invasive microbes can also shift the community structure and function of native microbial communities, which can have cascading downstream effects [4–7]. Increased globalization and human-mediated dispersal have significantly expanded microbial ranges at the global scale [8–11]. However, predicting invasion outcomes is stymied by a lack of knowledge about both life history of most invasive microbes and the invasibility of naive environments [12].

**Figure 1.**
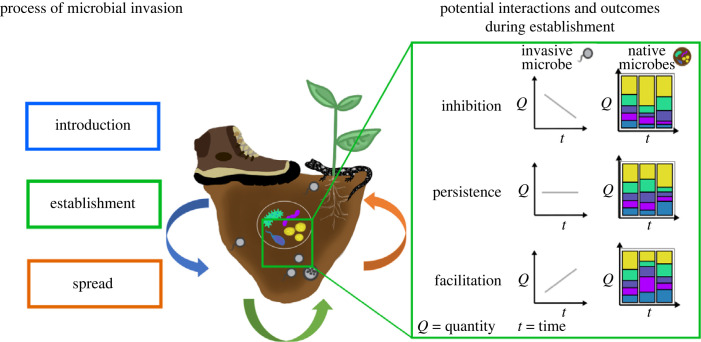
The microbial invasion process (modified from [14,15]) in the context of terrestrial invasion of an amphibian-killing fungal pathogen. Coloured arrows correspond to the steps of invasion. The grey microbe represents a pathogenic chytrid zoospore as it transitions through each phase of the process. A zoospore is introduced to a new terrestrial environment via human movement (blue arrow). The pathogen establishes a population and grows via reproduction (note depiction of zoosporangium, or the chytrid reproductive body, in this phase; green arrow). Zoospores are spread to potentially susceptible hosts (i.e. salamanders) and/or dispersed long distances (orange arrow). Our study focuses on experimentally evaluating a snapshot of this process, establishment (inset green box), and determining invasion outcomes.

Delineating factors influencing microbial invasion into and within environmental reservoirs can be challenging due to complexities prevalent in the invasion process. Generally, invasion stages are characterized by the introduction, establishment, and spread of alien species in a non-native environment [13] (figure 1). Each step presents specific challenges to successful infiltration; however, spread dynamics reveal that environmental invasibility by non-native microbes depends on the native microbial community composition and propagule pressure of the invading microbe [16–18]. Outcomes of invasion include inhibition of the invading species with no resulting establishment [19], persistence of the invader where establishment of a reproducing population may or may not occur over time [20], and facilitation where the invader is able to successfully establish and form a stable population [14]. Ultimately, each potential outcome of invasion should produce a response in the native microbial community due to indirect or direct interactions. Therefore, testing the invasion potential of a community while accounting for native microbes is pivotal for understanding and predicting invasion outcomes (figure 1).

Identifying the mechanisms by which microbes can invade novel environments is especially important in the context of emerging infectious diseases. For example, fungal diseases pose a substantial threat to life on Earth [21–23]. These emerging diseases have already jeopardized entire crop systems, decimated forests, influenced the health of millions of humans, and caused profound losses to global biodiversity, with trends showing increasing effects [23]. For amphibians, a global pandemic is occurring due to an infectious fungal disease known as chytridiomycosis [24], caused by the pathogens *Batrachochytrium dendrobatidis* (*Bd*) and *B. salamandrivorans* (*Bsal*), which has driven the largest declines of any taxa attributable to a single disease in history [25–27]. The ‘salamander-devouring fungus', *Bsal*, is an invasive pathogen that originated in Asia and has caused mass declines of fire salamander (*Salamandra salamandra*) populations in parts of Europe [28–30]. However, *Bsal* has not been detected in North America, the global hotspot of urodelan biodiversity [31,32]. The southeastern United States, in particular, is a region with high host endemism and favourable bioclimatic conditions for the fungus to grow, creating an optimal opportunity for unprecedented disease spread [33]. In addition, the genomic architecture of *Bd* and *Bsal* displays plastic genetic traits characteristic of invasive microbes [12,34], giving them an adaptive advantage in variable environments.

Potential inter- and intra-continental spread of *Bsal* may occur rapidly [33,35], especially in localities at the confluence of salamander biodiversity and anthropogenic influence. Evidence suggests dispersal rates of human-associated microbes have increased, particularly between Europe and North America [9]. Humans have facilitated the inter-continental spread of invasive fungal pathogens before. For example, *Pseudogymnoascus destructans*, the fungus that causes white-nose syndrome in bats, was most likely introduced from Europe to the eastern United States by people visiting caves in both regions across a short period [36]. *P. destructans* has since spread cross-continent to the Western US and has impacted over 6.7 million North American hibernating bats [37–39]. If *Bsal* were introduced to North America, salamander populations would likely experience similar severe impacts from chytridiomycosis, as many species in the United States are susceptible to infection under experimental conditions [40,41]. Less than 1 s of contact between hosts is sufficient for *Bsal* transmission in the highly susceptible and widely distributed newt, *Notophthalmus viridescens* [42]. Additionally, *Bsal*'s sister pathogen, *Bd*, is an introduced but now enzootic pathogen in North America [43], where it has caused population declines in anurans [44]. Co-infections of chytrids or other amphibian pathogens could compound disease-driven declines [45,46]. In addition, environmental reservoirs (e.g. soil [30], vegetation [47], and water [48]) facilitate the spread of *Bd* and *Bsal*. Therefore, determining the invasion potential of pathogenic chytrid fungi in the environments of naive hosts is paramount to establishing invasibility thresholds. Focusing on environmental invasibility can aid in planning mitigation strategies for areas of high conservation priority. Because of the risk of accidental introduction during experimentation, microcosm assays provide an excellent opportunity to investigate invasibility and can provide insights into the biotic and abiotic factors affecting microbial establishment.

Here, we investigated the potential for *Bsal* and *Bd* to establish and grow in areas with increased invasion risk due to human movement and high abundance of susceptible salamander hosts. We used experimental microcosms with soil collected from five locations throughout Great Smoky Mountains National Park to examine the ability of chytrid fungal pathogens to establish in these environments. We used long-read next-generation amplicon sequencing to characterize the response of the native soil microbial community to chytrid invasion. We hypothesized that: (i) *Bd* and *Bsal* would similarly establish and grow in the North American soil environment, (ii) higher pathogen propagule pressure would increase the invasibility of the soil environment, and (iii) soil bacterial communities would influence invasion similarly across site localities. Understanding invasibility dynamics will provide insight on establishment potential should *Bsal* be introduced to North America and help develop strategies to mitigate fungal pathogen impacts on native amphibian hosts.

## 2. Material and methods

### (a) Soil sample collection

**Figure 2.**
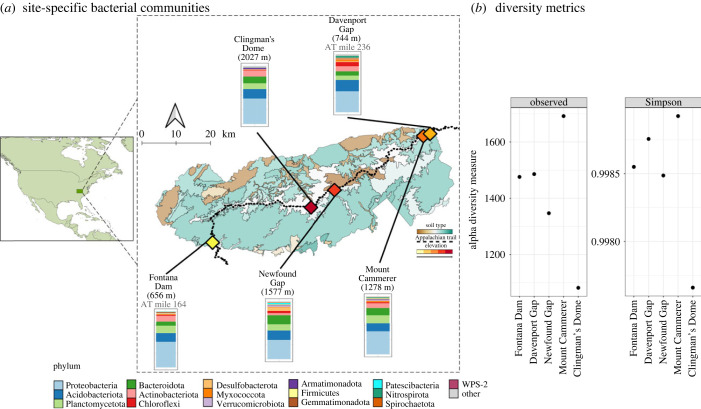
Comparison of bacterial alpha diversity metrics among sites. (*a*) Expanded insert shows the Great Smoky Mountains National Park location within North America (smaller map). Coloured portions of map within dashed lines indicate different underlying soil types. Soil names and the colour scheme are detailed in the electronic supplementary materials (electronic supplementary material, table S2). Dotted line bisecting the park indicates the Appalachian Trail. Diamonds correspond to soil sampling locations with a colour gradient related to elevation, where darker colour symbols are higher elevation sites. Stacked bar plots show the relative abundance of the initial bacterial community composition at the level of phylum for each site pre-invasion. Elevation values in metres are listed under each site name and Appalachian Trail mile values are listed for sites Fontana Dam and Davenport Gap to highlight relative distance values. (*b*) Observed number of amplicon sequence variants (ASVs) and Simpson diversity plots for soil samples pre-invasion.

In July 2020, we collected soil samples from five locations within Great Smoky Mountain National Park (figure 2, electronic supplementary material, table S1). Sites were adjacent to the Appalachian Trail in areas of frequent human foot traffic and within salamander habitat. We chose sites with different elevations, soil types, and that were distributed across the park area [49,50]. Newfound Gap and Clingman's Dome sites are more heavily trafficked by short-term visitors and day hikers while the other sites are more likely to be trekked by long-distance recreationalists with relatively less frequent human activity. Soil make-up and consistency ranged from loose particles (Fontana Dam) with a light texture (Clingman's Dome) to dense soils (Newfound Gap) and a high leaf litter content (Mt Cammerer, Davenport Gap). Regardless of soil type, we observed many individuals belonging to salamander genera such as: *Desmognathus*, *Eurycea*, *Notophthalmus* and *Plethodon* at or near sampling sites. Using a metal trowel, we filled four sterile Whirl-Pak bags (Whirl-Pak, Madison, WI, USA) with 100 g of soil from the O horizon at each site, for a total of 400 g of soil per location. We placed samples in an insulated cooler containing ice with bags separated from ice by a thin towel to keep samples cold but not frozen. Samples were transported to the laboratory at the University of Florida and kept at 4°C until processing. Because we collected multiple bags of soil from each sampling location (four Whirl-Pak bags of 100 g each), the total soil for each site was placed in sterilized plastic containers and homogenized to account for bag effects. All equipment was sanitized between sites using Zep disinfectant spray (Zep, Atlanta, GA, USA).

### (b) Experimental microcosm growth assays

We investigated *Bsal* and *Bd* growth response in soil samples from the Appalachian region of North America using *in vitro* microcosm growth assays. We autoclaved half of the soil from each site in glass beakers at 121°C for 30 min to serve as a microbially inactive control. For each experimental microcosm, we aliquoted 2 g of soil (sterilized and non-sterilized) into each well of a 12-well culture plate (Corning Costar, Corning, NY, USA) under a laminar flow hood. We set up one plate for each of four-time points (days 1, 4, 7 and 14) over a two-week period for each site (× 5 sites) with triplicate microcosms for each treatment combination of pathogen type, pathogen concentration and soil sterility. In total, we created 88 plates of sterile and non-sterile soil from each site for each time point and pathogen with pathogen concentration distributed in triplicate into the wells within each plate (electronic supplementary material, figure S1).

To inoculate the soil with fungal pathogens, we grew *Bsal* and *Bd* in culture plates until enough zoospores were present to produce the volume required for two different concentrations. *Bsal* (isolate no. AMFP1) was cultured on half-strength tryptone–gelatin hydrolysate–lactose (TGhL) media plates at 15°C following Robinson *et al*. [51]. *Bd* (ALKL1) was cultured on tryptone media at room temperature. Pathogen culture plates were flooded with DNase/RNase-free water (Invitrogen, MA, USA) and left for 30 min to allow for zoospore release. Liquid from each plate was then collected in a Falcon tube and filtered using a 10 μm syringe filter (catalogue no.: SF18127, Tisch Scientific, OH, USA). Zoospores were counted via haemocytometer. We prepared two concentrations of each pathogen to simulate varying levels of propagule pressure, then heat killed half of each zoospore solution by boiling at 100°C for 10 min. We pipetted 1 ml of each treatment solution into its corresponding soil aliquot, and the pipette tip was used to homogenize the solution distribution in each well. Control plates consisted of 1 ml of broth (*Bsal*, 1 : 2 TGhL; *Bd* 1% T) and 1 ml of inoculant. Within each plate, the following layout was created: inoculation with a lower concentration of live pathogen (*n* = 3), inoculation with a higher concentration of live pathogen (*n* = 3), inoculation with a lower concentration of heat-killed pathogen (*n* = 3), inoculation with a higher concentration of heat-killed pathogen (*n* = 3). Across plates, the following treatment combinations were performed: non-sterile soil with *Bsal* 10^3^, non-sterile soil with *Bsal* 10^4^, sterile soil with *Bsal* 10^3^, sterile soil with *Bsal* 10^4^, non-sterile soil with *Bd* 10^3^, non-sterile soil with *Bd* 10^5^, sterile soil with *Bd* 10^3^ and sterile soil with *Bd* 10^5^ (electronic supplementary material, figure S1). We selected pathogen concentrations based on culture spore production and at levels similar to or lower than concentrations used in other experiments [30].

Both pathogens were kept at the same temperature (15°C) to ensure consistency in a Percival incubator (model: I-41VL, Perry, IA, USA) on a 12 : 12 light : dark cycle. We grew both pathogens at the optimal growing temperature for *Bsal* because *Bd* is already established in North America. In addition, we focused on invasion potential of *Bsal* while also investigating the performance of *Bd* under identical conditions. Plates were removed at predetermined time points over the two weeks and sampled for microbial community and pathogen growth. To determine relative microbial activity in soil samples across time, we conducted live and dead cell staining at each time point using the LIVE/DEAD Sperm Viability Kit (catalogue no.: L7011, Invitrogen, MA, USA, see electronic supplementary materials for detailed methods).

### (c) DNA extraction and qPCR

Before sampling, we opened plates in a laminar flow hood and homogenized soil microcosms using sterilized metal scoopulas. Total genomic DNA (gDNA) was extracted using a DNeasy PowerSoil Pro kit (QIAGEN, Germantown, MD, USA) following the manufacturer's protocol with an input of 0.25 g of soil per microcosm. We sampled liquid media (broth) controls by mixing the solution briefly with a clean pipette tip and aliquoting 1 ml into sterile 1.5 ml microcentrifuge tubes. We centrifuged tubes for 3 min at 14 000 RPM to concentrate cells. The supernatant was discarded, and pellets were added directly to power bead tubes for extraction. UltraPure DNase/RNase-Free distilled water (Invitrogen, MA, USA) and ZymoBIOMICS Microbial Community DNA Standard was also extracted and included in sequencing as controls (Zymo Research, Irvine, CA, USA). We eluted 50 μl to be used in downstream analyses, and DNA extracts were kept at −20°C after elution. Post extraction, the amount of *Bsal* and *Bd* in each microcosm was quantified using quantitative PCR (qPCR). Reactions were run separately with pathogen-specific probes and standards of 0.1, 1, 10, 100 and 1000 zoospore genomic equivalents (ZGE). Due to high amounts of DNA recovered from soil extracts, samples were diluted 1 : 10 before running qPCR for optimal results and to reduce potential inhibition.

### (d) Metabarcoding using full-length 16S rRNA sequencing

We characterized soil bacterial communities and their response to chytrid fungi using 16S amplicon sequencing. DNA template was quantified using a Qubit 4 fluorometer (Invitrogen, MA, USA) and normalized using 10 mM Tris-HCL pH 8.0–8.5 prior to amplification. We concentrated low-yield samples using a SpeedVac (ThermoFisher Scientific, USA). We then amplified full-length 16S rRNA gene regions using barcoded primers following the ‘Amplification of Full-Length 16S Gene with Barcoded Primers for Multiplexed SMRTbell^®^ Library Preparation and Sequencing’ protocol (Pacific Biosciences, Menlo Park, CA, USA) for 23 cycles. Amplification products were visualized on a 1.2% agarose gel electrophoresis, pooled, and purified using AMPure PB beads (Pacific Biosciences, Menlo Park, CA, USA) before library construction. SMRTbell adapters were then ligated onto purified PCR products using SMRTbell Express Template Prep Kit 2.0 (Pacific Biosciences, Menlo Park, CA, USA). Libraries were sequenced on a PacBio Sequel IIe system at the University of Florida's Interdisciplinary Center for Biotechnology Research (ICBR). We deposited demultiplexed reads and associated metadata in the National Center for Biotechnology Information Sequence Read Archive under BioProject ID: PRJNA897848.

### (e) Sequence processing

We processed sequence data in R v. 4.1.0 [52]; first filtering reads less than 1000 basepairs (bp) and greater than 1600 bp. Filtered reads were further processed using the DADA2 pipeline [53] with default settings for PacBio reads (v. 1.20.0, following tutorial https://benjjneb.github.io/LRASManuscript/LRASms_fecal.html), providing a table of amplicon sequence variants (ASVs). Taxonomy was assigned using the Silva database v. 138.1, accessed on 17 May 2022 [54]. Sequence and taxonomy data were combined using the package *phyloseq* [55], under which we conducted microbial community analyses. To identify and remove any bacterial contaminants, we ran our data through the *decontam* R package [56], assigning broth, sterilized soil samples and laboratory controls (e.g. PCR water control) as ‘controls’ with all others assigned as ‘true samples’. Contaminant ASVs were removed from the final dataset (electronic supplementary material, table S3). In total, the mean number of reads were 10 940 (338–19 084). Analyses were performed with raw reads as the dataset rarefied to a depth 90% of the minimum sample showed similar results when comparing commonly used diversity metrics (electronic supplementary material, figure S2) [57]. Here we present outcomes of the non-rarefied dataset [58] and refer readers to our code for reproducible results (see Data accessibility).

### (f) Statistical analyses

We examined differences in alpha diversity, specifically observed ASVs and Simpson's diversity measurements, using Kruskal–Wallis and subsequent Dunn's tests with a Benjamini–Hochberg stepwise adjustment at a significance threshold of 0.05. We used this correction to reduce the rate of false positives inherent in multiple hypothesis testing among pathogens and sites [59]. We visualized the relative abundance of bacterial taxa within samples using the *microViz* R package [60]. Beta diversity across time and between sites was tested with permutational multivariate analysis of variance (PERMANOVA) using the non-parametric *adonis* function from the *vegan* R package [61] with pairwise PERMANOVAs using the *pairwise.adonis2* from the *pairwiseAdonis* package [62]. Differences in the pathogen load and the number of live and dead cells across days post-pathogen introduction were also determined using Kruskal–Wallis, with subsequent Dunn's tests as necessary, with a threshold of 0.05. Mann–Whitney *U*-tests were used for comparisons of only two groups. Prior to analyses of pathogen load and cell count data, we eliminated outliers that might skew results by removing samples that significantly deviated from two of the three replicates. Biomarker analysis of the bacterial community response to pathogen introduction was performed using the *microbiomeMarker* R package [63] and the *run_lefse* function [64], with linear discriminant analysis (LDA) scores greater than 2.0 considered significant. We then used basic local alignment search tool (BLAST) to compare our sequence data against bacteria in the Antifungal Isolates Database [65] with the software Geneious Prime v. 2022.2.1 using a threshold of 100% sequence identity.

## 3. Results

### (a) *Bd* and *Bsal* response to microbially active and inactive soils

Quantitative PCR results showed that the amount of *Bd* zoospores decreased from day 1 to 14 in all non-sterile treatments, except in broth controls, as we expected (Mann–Whitney tests: 10^3^ *W* = 208, *p* < 0.05; 10^5^ *W* = 210, *p* < 0.05). *Bd* zoospore amounts remained unchanged for sterilized soils (Mann–Whitney: 10^3^ *W* = 120, *p* = 0.53; 10^5^ *W* = 140, *p* = 0.26) and in broth controls (Mann–Whitney: *W* = 55, *p* = 0.524) (figure 4*a*). Similarly, *Bsal* zoospore quantities decreased between days in non-sterile soil (Kruskal–Wallis: 10^3^ *χ*^2^ = 18.97, *p* < 0.05, d.f. = 2; 10^4^ *χ*^2^ = 17.19, *p* < 0.05, d.f. = 2) (figure 4*b*). However, pairwise comparisons using Dunn's test indicated declines occurred after day 1 (Dunn's: comparisons 1–7 and 1–14, adjusted *p*-value < 0.05) but not between days 7 and 14 (7–14, adjusted *p*-value > 0.05). Sterilized soils inoculated with *Bsal* responded differently based on propagule size. Control soils inoculated with 10^3^ concentration showed no difference in *Bsal* zoospore quantity between days (Kruskal–Wallis: *χ*^2^ = 0.608, *p* = 0.73, d.f. = 2), while soils inoculated at 10^4^ concentration were significantly different across time (Kruskal–Wallis: *χ*^2^ = 10.88, *p* = 0.004, d.f. = 2). The first day differed from other time points (Dunn's test: comparisons 1–7 and 1–14, adjusted *p*-value < 0.05) but day 7 and 14 did not significantly differ (Dunn's test: comparison 7–14, adjusted *p*-value = 0.428). *Bsal* broth controls showed an unexpected decrease in zoospore detection over time, with differences found between all time points (Kruskal–Wallis: *χ*^2^ = 22.681, *p* < 0.05, d.f. = 2; Dunn's test: all comparisons, adjusted *p*-value < 0.05). Live/dead cell counts, a proxy of microbial activity, were not significantly different across time within treatments; however, as predicted, the number of live cells was much higher in non-sterilized soils compared to the sterilized controls (*Bd*: Kruskal–Wallis: *χ*^2^ = 27.238, *p* < 0.05, d.f. = 3; Dunn's test: all non-autoclaved – autoclaved comparisons, adjusted *p*-value < 0.05; *Bsal*: Kruskal–Wallis: *χ*^2^ = 32.121, *p* < 0.05, d.f. = 3; Dunn's test: all non-autoclaved – autoclaved comparisons, adjusted *p*-value < 0.05). Dead cell counts did not differ across time or between active and inactive soils (figure 4).

### (b) Soil bacterial communities differentially shifted upon amphibian fungal pathogen invasion

**Figure 3.**
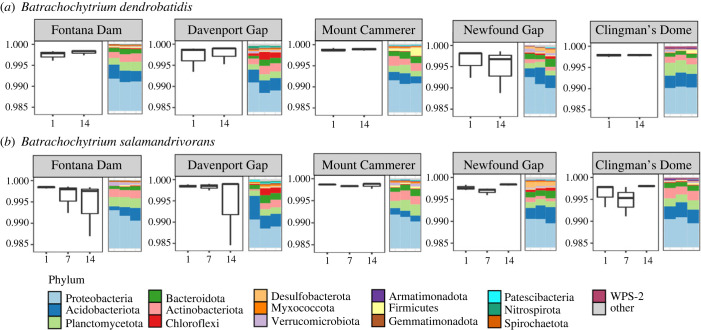
(*a*) Simpson's alpha diversity boxplots for soil bacterial communities inoculated with *Bd* by day for each site. Stacked bar plots correspond to day 14 samples (triplicate) demonstrating bacterial community composition post-invasion. (*b*) Simpson's diversity boxplots and day 14 stacked bar plots for soils inoculated with *Bsal*.

We identified 36 648 ASVs from 80 soil samples before adding chytrid pathogens and across the two weeks after pathogen inoculation. Overall, the phylum Proteobacteria was the most abundant (40.75%), followed by Acidobacteria (16.51%), Planctomycetota (11.73%), Actinobacteriota (10.10%) and Bacteroidota (8.10%). Pre-inoculated soils had slightly higher Bacteroidota (11%) than Actinobacteriota (8.2%) (figure 2*a*). Post-inoculation and after the two-week incubation, relative abundance levels remained similar for Proteobacteria (39%), Acidobacteria (17%) and Plactomycetota (11%), but increased in Actinobacteriota (10%) abundance and decreased for Bacteroidota (8%) for both pathogens (figure 3*a,b*). Mock community standard results matched manufacturer estimates.

Bacterial communities remained similar over time for observed (Kruskal–Wallis: *χ*^2^ = 3, *p* = 0.39, d.f. = 3) and Simpson diversity estimates (Kruskal–Wallis: *χ*^2^ = 4.61, *p* = 0.2, d.f. = 3). However, bacterial community richness and dominance significantly contrasted among sampling sites (observed – Kruskal–Wallis: *χ*^2^ = 9.81, *p* = 0.04, d.f. = 4; Simpson – Kruskal–Wallis: *χ*^2^ = 21.22, *p* = 0.0002, d.f. = 4; figure 2*b*). Pairwise analyses revealed that sites Newfound Gap and Clingman's Dome have significantly different dominant bacterial communities from Mount Cammerer and Davenport Gap (Dunn's test: adjusted *p*-value < 0.05) but similar richness (Dunn's test: adjusted *p*-value > 0.05). Pathogen-specific comparisons showed no notable differences in community richness. Dominance diversity estimates significantly differed by site for *Bsal* inoculated soils (Simpson – Kruskal–Wallis: *χ*^2^ = 12.32, *p* = 0.01, d.f. = 4) with the highest elevation site, Clingman's Dome, showing less dominant bacterial communities compared to Mount Cammerer (Dunn's test: adjusted *p*-value = 0.03) (figure 2*b*) and generally for all sites (figure 3*a,b*). Overall, the Mount Cammerer and Davenport Gap sites had the highest bacterial richness and dominance (figure 2*b*). Beta diversity estimates of Bray–Curtis distances (presence and abundance of taxa) showed that bacterial communities did not significantly shift across days (Adonis test: *F*_(1,78)_ = 0.512, *p* = 0.36). However, we detected an effect of site (pairwise Adonis: *p* < 0.05 for all comparisons) with slight shifts across time (electronic supplementary material, figure S3).

### (c) Bacterial biomarkers of inhibition against amphibian-killing fungi

From the LDA analysis, we found 20 ASVs with relative abundances significantly higher on day 14 than day 1 in at least one site for both *Bd* and *Bsal* (figure 5 taxa in boldface). The taxon most closely identified belonging to genus *Burkholderia-Caballeronia-Paraburkholderia* had the highest abundance levels and was significant across all five sites. While at most sites it had higher abundance on day 1 than day 14, the highest elevation site, Clingman's Dome, showed an increase in bacterial abundance over time in response to both pathogens. Species *Bradyrhizobium erythrophlei* was the only other ASV showing significant change across all sites, but at lower abundance than *Burkholderia-Caballeronia-Paraburkholderia*. Twelve of the 20 ASVs are known from soil and terrestrial habitats while five are associated with aquatic habitats. Mid-elevation sites had the highest number of significant ASVs with the lowest elevation sites, Fontana Dam and Davenport Gap, having the least. Site Mount Cammerer had the most ASVs and the highest abundances (figure 5). Of amphibian-associated bacteria tested for their antifungal properties [65], 3% of our sequences matched at 100% sequence identity, with seven closely matching bacteria having known inhibitory function against *Bd* (electronic supplementary material, table S5). We found two sequences that were 100% similar to Burkholderiaceae bacterium in the genus *Delftia*. Also, three sequences matched inhibitory bacteria in the genus *Pseudomonas,* and one to an inhibitory *Bacillus* bacterium.

## 4. Discussion

Environmental reservoirs such as soils serve as a cryptic and understudied contributor to the establishment of the invasive fungal pathogens that cause the disease chytridiomycosis in amphibians [44,47,66]. We found that invasibility of soil communities in Great Smoky Mountains National Park varied with the introduction of the different fungal species. Overall, soil environments did not facilitate pathogen invasion in experimental microcosms over the two-week incubation period. However, *Bd* and *Bsal* responded differently within the soil environments, with *Bd* being more inhibited across sites and demonstrating the effects of propagule pressure. While the incubation temperature used in this experiment was outside of the known optimal growth range for this pathogen (17–25°C)[67], *Bd* growth still occurs at 15°C and site-specific differences suggest inhibitory processes outside of temperature effects. However, we recommend future studies incorporate varied temperatures. *Bsal* persisted across all time points and sites regardless of active microbial community presence or concentration. Together, these results demonstrate a need to identify factors influencing invasion dynamics on a per-species basis. Additionally, we recommend evaluation of specific strains due to pathogen evolutionary dynamics that may affect establishment potential [68].

### (a) Barriers to the establishment of amphibian-killing fungi in soil

**Figure 4.**
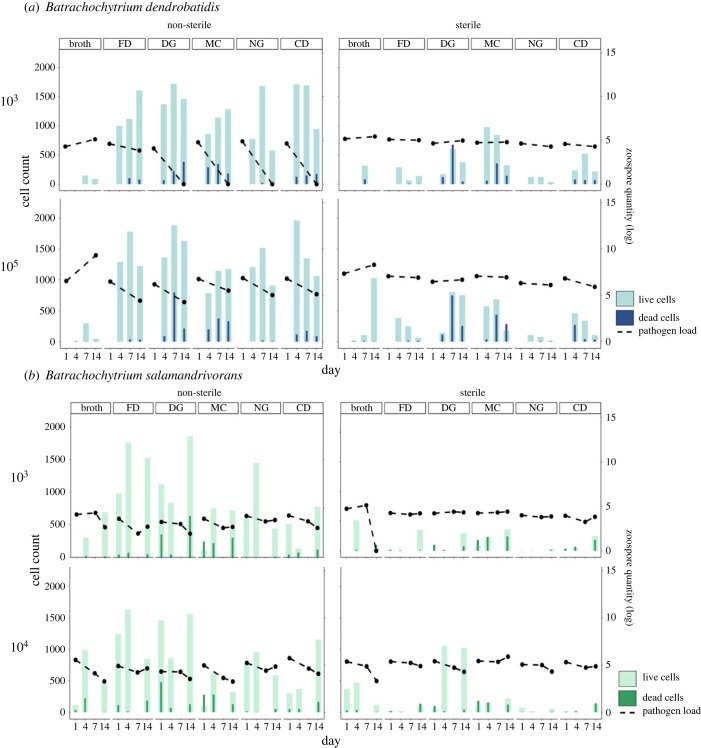
Soil microbial community responses to chytrid fungal pathogens over time. Chytrid pathogen load corresponding to qPCR results is represented as the log 10 zoospore quantity and is shown on the right-hand *y*-axis. Microbial activity corresponding to live and dead cells counts is represented by the coloured bar plots and are shown on the left-hand *y*-axis. (*a*) *Bd* inoculated soils and (*b*) *Bsal* inoculated soils. Columns denote non-sterilized soils and sterilized soil controls while rows correspond to propagule pressure as varying pathogen concentrations.

We did not observe increased abundances of *Bd* and *Bsal*, indicating barriers to proliferation in experimental microcosms (figure 4). Patterns of pathogen response to invasion for *Bd* showed greater inhibition compared to *Bsal*, although this relationship decreased with increased propagule pressure (figure 4*a*). The lower concentration of *Bd* zoospores, or propagules, were completely inhibited in soils from three sites but not in sterile controls (figure 4*a*), indicating that natural soil biotic communities at these locations differentially influence *Bd* terrestrial establishment [69–71]. Soil bacterial communities were significantly different between sites, particularly locality-specific dominant taxa (figure 2), adherent to known terrestrial microbial patterns related to soil composition and distance [72,73]. However, the highest elevation site inhibited *Bd* growth and showed a negative trend for *Bsal* growth (figure 4*a,b*) while having the lowest overall bacterial diversity and dominance (figure 2*b*), contrary to the pattern that more diverse communities are more resistant to invasion [17,18]. Other sites showed the greatest variability in bacterial dominance across time in response to *Bsal* invasion but not *Bd* (figure 3). While examining abiotic soil parameters and microbial inhibition mechanisms was outside this study's scope, research examining how elevation and terrestrial properties drive soil microbial assembly will identify potential mechanisms of environmental resistance in these habitats. We hypothesize that predation by micro-predators [74], competition or decomposition from acidophilic bacteria [75] and native soil fungal communities [69,71] are potential factors inhibiting non-native fungal invasion. Additionally, because we did not recover any *Bd* DNA on day 14 for most of the sites, this result suggests decomposition, but not competitive exclusion, may be a dominant barrier for *Bd* in these environments [74,76,77]. Potential drivers of decomposition could be anti-*Bd* metabolites [65], mycophagous bacteria or fungi [78], and other micro-predators such as microeukaryotes [79,80]. We suggest future studies should expand on multi-omics approaches to help explain fungal decomposition, including metabolomics [81], fungal community ITS metabarcoding [82] and eukaryotic 18S metabarcoding [83]. Although *Bsal* persisted at relatively stable levels in non-sterilized and sterilized soils compared to *Bd* (figure 4*b*), investigating methods of natural suppression are worthwhile to potentially combat both propagule effects and pathogen-specific differences.

### (b) Identification of anti-batrachochytrid biomarkers in soil

**Figure 5.**
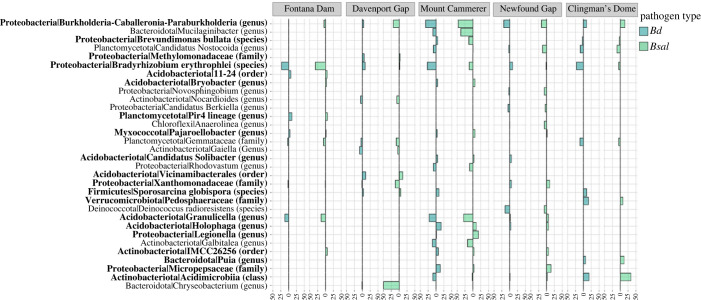
Amplicon sequence variants (ASVs) that significantly changed in mean relative abundance across time points based on linear discriminant analysis (LDA). Values represent the difference in mean abundances between days 14 and 1. Taxa names on the *y*-axis include phylum and the highest level of taxonomic resolution possible. Taxa in bold had a higher relative abundance of ASVs on day 14 for either *Bd* or *Bsal* in at least one site. Locality names at the top are in order of elevation from lowest to highest.

While native soil bacteria communities differed between sites (electronic supplementary material, figure S3), we found specific ASVs that increased significantly in response to pathogen introduction (figure 5). From the list of 20 (electronic supplementary material, table S4), one of the most promising as a potential anti-batrachochytrid biocontrol was genus *Burkholderia-Caballeronia-Paraburkholderia.* This taxonomic designation includes the names of three genera likely due to taxonomic controversies or confusion surrounding assignments for these sequences; therefore, we refer to the higher-order name, Burkholderiaceae. This family is within the phylum Proteobacteria, which constitutes the majority of bacterial taxa found in soils from this study (figures 2*a*, and 3) and is also a significant component of skin microbiomes of amphibian hosts [84–86]. Within this family, bacteria in the genus *Paraburkholderia* are a diverse group of environmental microbes that form close associations with plant and fungal tissues [87–89]. Recent taxonomic work has reclassified many *Paraburkholderia* species to the *Burkholderia* genus [87,90]. *Burkholderia* species are known to produce a wide variety of antimicrobial metabolites targeting microbial pathogens [87,90–92], many of which are potent against fungal pathogens of plants [93–95], humans [96], and an invertebrate-symbiotic fungus [97]. Some Burkholderiaceae taxa do carry anti-batrachochytrid functions [65,84]. Therefore, we recommend these bacteria for further research into *Bd* and *Bsal*-specific antifungal properties, which may aid in future mitigation efforts. In addition, soil microbial communities should be surveyed for antifungal isolates in the same manner that has been done from amphibian skin [65,84]. Characterizing environmentally isolated anti-chytrid isolates would increase our understanding of how soil microbial communities limit invasive species, adding candidates to control these pathogens. The differential responses in *Bd* and *Bsal* persistence in the North American soils we observed makes testing antifungal isolates on *Bsal* inhibition particularly important [85,98].

### (c) Pathogen persistence across sites at the Great Smoky Mountains National Park

*Bsal* persisted in the soil regardless of active microbial community presence, even though zoospore load diminished in nutrient media controls (figure 4*b*). Contrasting to the dynamics of *Bd*, both concentrations of *Bsal* remain stable over time. Viable propagules present in terrestrial environments, even in the absence of active growth, are enough to initiate new infections [30,99,100]. These findings, although not surprising, suggest that *Bsal* persistence in the North American terrestrial environment could lead to infection in naive North American hosts. For this study, we filtered chytrid cultures for flagellated, motile zoospores to inoculate soil, but the *Bsal* life cycle includes an additional encysted zoospore stage [30]. Encysted fungal spores are non-motile and have a thicker cell wall to persist during harsh environmental conditions [30,101]. In addition, isolate-specific adaptations for saprotrophic traits allow some *Bsal* strains to obtain their nutrients from the environment, reducing the reliance on host density for transmission [68]. The persistence of *Bsal* in this study is troubling as we predict encysted spores will be able to remain viable in the terrestrial environment over extended periods, which increases the likelihood of potential host infection and long-distance dispersal events. The persistence of pathogens, including their components such as extracellular DNA post-mortality [102], increases interactions with native microbes which can lead to antimicrobial resistance [103] and/or horizontal gene transfer [104], expanding potential negative consequences [105]. While we did not explicitly test pathogen viability at the end of this experiment, we suggest that future experiments should include use of live animals and test infection rate post pathogen positive environmental exposure at varying time intervals, such as in Stegen *et al*., [30].

Experimental microcosms are an integral step in determining environmental invasibility to non-native microbiota [15], including emerging pathogens. The North American soil environment within Great Smoky Mountains National Park resisted invasion by *Bd*, but as propagules increased, this effect lessened. *Bsal*, which did not grow in experimental microcosms, showed greater persistence and a higher propensity for establishment. The experiment was limited to two weeks post-introduction; therefore, we recommend that future studies extend past this point to establish how long *Bsal* can persist and what its thresholds for environmental reproduction are, particularly in relation to varying climatic conditions and seasonality. It is suggested that pathogens will increase in relative abundance under future warming climate projections [106,107] and their environmental reservoirs remain understudied with regards to climactic variability [105]. Research into environmental invasibility of pathogens should consider climate change conditions and increased extreme weather intensification. We also recommend that future research focus on the co-invasion of different environments since *Bd* has a global distribution and *Bsal* is likely to spread [44,101,108]. Multiple introduction events could alter the native microbial community enough to make future establishment events more likely [5]. Inversely, mitigation efforts, including the use of chemical disinfectants [109,110] or environmental bioaugmentation [111], which are useful tools to combat pathogen invasion and disease effects, may disrupt natural microbial communities. Examining biotic community interactions in response to chemical or microbial alteration and across soil depths would be useful areas for future research, as perturbations could influence establishment and variation occurs between surface soil and deeper terrestrial microbial communities [112]. Determining environmental invasibility, microbe invasion potential and likely pathways of spread are crucial to developing better surveillance and management strategies for amphibian chytrids and other emerging pathogens. Holistically understanding the range of factors that influence microbial invasion is critical to implementing effective decontamination protocols, identifying priority regions of high invasibility and ultimately stopping the spread of invasive pathogens.

## Data Availability

The datasets generated during and analysed during the current study are available via the Dryad Digital Repository at https://doi.org/10.5061/dryad.rv15dv4g1 [113]. Raw sequences were deposited in the National Center for Biotechnology Information Sequence Read Archive under BioProject ID: PRJNA897848. Supplementary material is available online [114].
